# Draft Genome Sequence of *Bacillus alcalophilus* AV1934, a Classic Alkaliphile Isolated from Human Feces in 1934

**DOI:** 10.1128/genomeA.01175-14

**Published:** 2014-11-13

**Authors:** Oliver Attie, Anitha Jayaprakash, Hardik Shah, Ian T. Paulsen, Masato Morino, Yuka Takahashi, Issay Narumi, Ravi Sachidanandam, Katsuya Satoh, Masahiro Ito, Terry A. Krulwich

**Affiliations:** a Department of Genetics and Genomic Sciences, Icahn School of Medicine at Mount Sinai, New York, New York, USA; b Department of Chemistry and Biomolecular Sciences, Macquarie University, Sydney, NSW, Australia; c Department of Pharmacology and Systems Therapeutics, Icahn School of Medicine at Mount Sinai, New York, New York, USA; d Graduate School of Life Sciences, Toyo University, Itakura, Gunma, Japan; e Bio-Nano Electronics Research Centre, Toyo University, Kawagoe, Saitama, Japan; f Ion Beam Mutagenesis Research Group, Medical and Biotechnological Application Unit, Quantum Beam Science Center, Japan Atomic Energy Agency, Takasaki, Gunma, Japan

## Abstract

*Bacillus alcalophilus* AV1934, isolated from human feces, was described in 1934 before microbiome studies and recent indications of novel potassium ion coupling to motility in this extremophile. Here, we report draft sequences that will facilitate an examination of whether that coupling is part of a larger cycle of potassium ion-coupled transporters.

## GENOME ANNOUNCEMENT

*Bacillus alcalophilus* AV1934 is among the earliest alkaliphilic bacteria reported, in 1934. Aron Vedder (1) noted that this new *Bacillus* species, which he isolated from human feces, grew in an unusually alkaline pH range; the accepted strain designation, AV1934, honors Vedder’s accomplishment. Recently, *B. alcalophilus* AV1934 became a focus of renewed interest, apart from ongoing studies of the alkali-adaptive features of its proteins (2, 3). First, its isolation from human feces raises the possibility that this alkaliphilic strain may occur as part of the human distal gut microbiome, where other alkaliphiles have been found (4, 5). Second, *B. alcalophilus* AV1934 exhibits the novel ability to couple flagellar motility with either inward potassium or sodium fluxes (6). Potassium coupling was a departure from the generalization that inwardly directed sodium ion gradients energize flagellar motility and ion-coupled solute uptake systems in alkaliphiles that grow at pH >9.5 (7, 8). We sought genomic data that would allow us to test whether the sodium-coupled bioenergetic cycles that usually support alkaliphile solute uptake and cytoplasmic pH homeostasis have an alternate potassium coupling version in *B. alcalophilus* AV1934. Such genomic information was not publically accessible, although a project has been registered in the National Center for Biotechnology Information (NCBI) database in 2005 (BioProject accession no. PRJNA13375). Therefore, we undertook a sequencing project of the extremely alkaliphilic *B. alcalophilus* AV1934 (ATCC 27647).

The draft genome sequence was generated by a Roche GS Junior, with 183,644 variable-length reads, resulting in 23-fold coverage of the genome. These were assembled by the Newbler 2.7 assembler to yield 182 large contigs (>500 bp) assembled from 182,815 reads. Annotation was done using the Prokaryotic Genomes Automatic Annotation Pipeline (PGAAP). The G+C content of the genome is 37.2%. The draft genome has 4,348,660 bp, with 3,745 predicted proteins. An earlier draft genome was completed, with 427 contigs and 4,237,661 bp, having been assembled with the Inchworm assembler, which is part of the Trinity package. For annotation, PGAAP was used, along with the program of Ren, Kang, and Paulsen (9). This draft genome has 4,095 predicted genes and 4,063 predicted proteins.

In addition to the *mot* genes encoding the MotPS channel that uses either potassium or sodium coupling (6), two loci encoding multisubunit Mrp-type antiporters (BalcAV_020445-020470 and BalcAV_211925-211955) in contigs ALPT02000010.1 and ALPT02000036.1, respectively, were found. This differs from the single essential sodium/proton Mrp antiporters of the alkaliphilic *Bacillus halodurans* C-125 and *Bacillus pseudofirmus* OF4 (10, 11). The *B. alcalophilus* AV1934 genome reveals no tripartite ATP-independent (TRAP-T family) uptake systems (12, 13), a major sodium-coupled complement in the other two alkaliphiles (14, 15). The *B. alcalophilus* AV1934 genomic data will enable us to test the hypothesis of a major role of potassium in ion coupling in this extremophile, as suggested for insect hind gut-associated bacteria (16).

### Nucleotide sequence accession numbers.

The whole-genome shotgun project has been deposited in GenBank under the accession no. ALPT02000000; it is the second draft version, which was used for gene descriptions in this paper. An earlier version noted in the paper has been deposited in GenBank under accession number no. ALPT00000000.
